# Long-Term Care Facilities: Important Participants of the Acute Care Facility Social Network?

**DOI:** 10.1371/journal.pone.0029342

**Published:** 2011-12-27

**Authors:** Bruce Y. Lee, Yeohan Song, Sarah M. Bartsch, Diane S. Kim, Ashima Singh, Taliser R. Avery, Shawn T. Brown, S. Levent Yilmaz, Kim F. Wong, Margaret A. Potter, Donald S. Burke, Richard Platt, Susan S. Huang

**Affiliations:** 1 Departments of Medicine and Biomedical Informatics, University of Pittsburgh, Pittsburgh, Pennsylvania, United States of America; 2 Department of Epidemiology, Graduate School of Public Health, University of Pittsburgh, Pittsburgh, Pennsylvania, United States of America; 3 Division of Infectious Diseases and Health Policy Research Institute, University of California Irvine, Irvine, California, United States of America; 4 Department of Health Management and Policy, Center for Public Health Practice, Graduate School of Public Health, University of Pittsburgh, Pittsburgh, Pennsylvania, United States of America; 5 Department of Population Medicine, Harvard Medical School and Harvard Pilgrim Health Care Institute, Boston, Massachusetts, United States of America; 6 Department of Biostatistics, Graduate School of Public Health, University of Pittsburgh, Pittsburgh, Pennsylvania, United States of America; 7 Pittsburgh Supercomputing Center, Carnegie Mellon University, Pittsburgh, Pennsylvania, United States of America; 8 Center for Simulation and Modeling, University of Pittsburgh, Pittsburgh, Pennsylvania, United States of America; 9 Graduate School of Public Health, University of Pittsburgh, Pittsburgh, Pennsylvania, United States of America; University of Maryland, United States of America

## Abstract

**Background:**

Acute care facilities are connected via patient sharing, forming a network. However, patient sharing extends beyond this immediate network to include sharing with long-term care facilities. The extent of long-term care facility patient sharing on the acute care facility network is unknown. The objective of this study was to characterize and determine the extent and pattern of patient transfers to, from, and between long-term care facilities on the network of acute care facilities in a large metropolitan county.

**Methods/Principal Findings:**

We applied social network constructs principles, measures, and frameworks to all 2007 annual adult and pediatric patient transfers among the healthcare facilities in Orange County, California, using data from surveys and several datasets. We evaluated general network and centrality measures as well as individual ego measures and further constructed sociograms. Our results show that over the course of a year, 66 of 72 long-term care facilities directly sent and 67 directly received patients from other long-term care facilities. Long-term care facilities added 1,524 ties between the acute care facilities when ties represented at least one patient transfer. Geodesic distance did not closely correlate with the geographic distance among facilities.

**Conclusions/Significance:**

This study demonstrates the extent to which long-term care facilities are connected to the acute care facility patient sharing network. Many long-term care facilities were connected by patient transfers and further added many connections to the acute care facility network. This suggests that policy-makers and health officials should account for patient sharing with and among long-term care facilities as well as those among acute care facilities when evaluating policies and interventions.

## Introduction

Previous studies have demonstrated that individual acute care facilities do not function in isolation, but instead are connected to each other by shared patients. These hospital social networks (similar to ones formed by people) can have numerous policy-making implications [1], [2]. Since hospitals transfer patients to and from one another, policies or conditions affecting patients in one hospital can affect other hospitals connected to that hospital by patient sharing. For example, outbreaks of infectious diseases in one hospital could spread to other connected hospitals [3]. Furthermore, social network analyses, which have traditionally been used for mapping relationships among people in a population, can help elucidate and analyze an acute care facility only network [2].

However, focusing solely on acute care facilities neglects a potential key player in the inpatient healthcare facility social network: long-term care facilities (LTCFs), or nursing homes, which send patients to and from acute care facilities. Acute care hospitals may not be cognizant of all the LTCFs to which their patients have been recently admitted, and vice versa. Even when an individual hospital may be aware of which LTCFs transfer patients to and from its facility, it may not comprehend the extent of these connections and how these connections may indirectly connect hospitals to each other. Understanding these connections can assist hospital and public health policy making by identifying which LTCFs (and their policies) may affect which hospitals. For example, the infection control and chronic disease management programs of a LTCF may affect the acute and long-term care facilities with which it is connected.

While previous studies have included only acute care facilities, our study sought to elucidate the patient transfer connections among all inpatient facilities (long-term and acute care) in Orange County (OC), CA, a large and diverse metropolitan county, utilizing social network principles, measures, and frameworks. The objectives of this study were to determine and characterize:

1. The number of patient transfers among LTCFs in Orange County.
2. The number of direct patient transfers among acute and LTCFs in Orange County.
3. The degree to which LTCFs connect, by patient transfers, acute care facilities that are otherwise not connected.
4. How patient transfers correlate with geographic distance (i.e., are LTCFs more likely to transfer to or receive from hospitals that are in close proximity)?

## Methods

### Acute and Long-term Care Facilities in Orange County, CA

Table 1 summarizes the characteristics of the inpatient healthcare facilities in OC. Our study used 2007 patient-level data for all inpatient admissions (adult and pediatric) from all 32 acute care facilities and 72 LTCFs in the county. These facilities serve a total population of 3.1 million people residing in 148 zip codes. Acute care facility data were obtained from the California Office of Statewide Health Planning and Development (OSHPD) [4], which mandates reporting of all hospitalizations in the state. Patient transfer data from LTCFs were obtained from LTCF surveys which requested the annual number of patient transfers (including short-term rehab beds) between various acute care facilities and the LTCF. Additional LTCF characteristics data came from OSHPD and the national Long Term Care Minimum Data Set [4], [5]. Of the 32 acute care facilities in OC, six are long-term acute care (LTAC) facilities, where patients with prolonged high-level medical care needs (e.g., chronic mechanical ventilation) are treated. Three of the 32 are children's hospitals, one of which is an LTAC.

**Table 1:** Healthcare Facility Characteristics.

Facility Characteristics	Long Term Care Facilities			Acute Care Facilities		
	Mean (SD)	Median	Range	Mean (SD)	Median	Range
**Annual Admissions**	504 (863)	311	3–7,080	10,171 (8,359)	8,768	101–32,931
**Licensed Beds**	107 (59)	99	9–300	198 (119)	198	114–282
**% Male**	35 (14)	31	14–90	43 (7)	40	33–59
**% White**	68 (25)	71	4–100	72 (19)	77	19–92
**% Black**	2 (2)	1	0–12	3 (4)	2	1–18
**% Asian**	15 (22)	7	0–96	10 (8)	8	0–44
**% Hispanic**	16 (14)	13	0–80	25 (18)	21	5–77

Data elements included demographics, diagnoses, procedures, and unique encrypted patient identifiers (based upon elements such as social security number) that remained consistent for each patient regardless of the admitting hospital [6]. The Institutional Review Boards of the University of California Regents and the California Committee for the Protection of Human Subjects approved this study; it was exempt from review at the University of Pittsburgh.

### Social Network Analyses

Social network analyses utilized UCINET for Windows, Version 6.311 (Analytic Technologies, Lexington, Kentucky). We created healthcare facility sociograms, where each node represented a healthcare facility and each edge (connection between two nodes) represented direct patient transfers. The sociograms arranged nodes in a circular pattern in order of decreasing bed capacity within strata, with hospitals ordered first, followed by LTACs, and ending with LTCFs. Node sizes were proportional to the number of licensed beds for each facility. Edges were directional, i.e., if Facility A sent patients to Facility B but did not receive patients from Facility B, then Facility B was connected to Facility A but not vice-versa (i.e., patient transfers are directed from A to B). Arrows indicated the direction of patient transfers, where a double-arrowed line connecting two facilities implied a symmetric connection. Sociograms were binary with an edge present if patient transfers volume (N) between two facilities exceeded a threshold number (N≥1 or N≥10). Our analyses considered three patient transfer networks: 1) acute care facilities only (i.e., hospitals and LTACs), 2) LTCFs only, and 3) both acute and long-term care facilities. Table 2 lists the social network measures that we calculated for the three networks.

**Table 2:** Social Network and Ego Network Measures Utilized.

	Description	Interpretation
**Social Network Measure**		
Number of Ties	Total number of inter-facility connections in the network	More ties = more interconnected
Density	Number of existing ties divided by the total number of possible ties in a network	Lower density = sparser network
Reciprocity	Number of facility pairs with bidirectional ties divided by the number of connected facility pairs	Lower reciprocity = more unidirectional ties
Geodesic Distance	Shortest number of inter-facility ties that connect one facility to another (i.e., shortest path needed to travel from one facility to another)	Smaller geodesic distance = fewer intermediaries between two facilities
Network Diameter	Largest geodesic distance in the connected network	Greater diameter = network less tightly connected
Betweenness	Number of times a given facility is part of the shortest path between two others (i.e., how often a given facility serves as an intermediary between other facilities)	High betweenness = facility serves as an intermediary between many pairs of facilities
Out-degree	Total number of different facilities that receive patients from a given facility	High out-degree = facility can affect many other facilities
In-degree	Total number of different facilities that send patients to the given facility	High in-degree = facility can be affected by many other facilities
**Ego Network Measure**		
Size	Number of other facilities directly connected to the ego facility	Larger size = more facilities an ego directly interacts with
Ties	Number of connections among all facilities in the ego network, excluding those involving the ego facility	Lower number of ties = fewer connections in ego network
Density	Ego network number of ties divided by the number of possible ties among the other facilities in the ego network	Lower density = ego is central player for connecting two facilities
Betweenness	Summed proportion of instances where the ego facility is part of the geodesic distance between two other facilities in its ego network (i.e., percent of all geodesic paths from neighbor to neighbor that pass through the ego)	Higher betweenness = ego is key player in establishing ties between facilities connected to it

To identify the acute and LTCF facilities with which each acute care facility most closely interacts, we characterized each facility's one-step ego network, which consisted of a facility (ego) and all other facilities directly connected to that ego (i.e., facilities that directly transferred patients to and from the ego), as well as the ties among all the facilities in the ego's network. Table 2 also lists the social network measures applied to each ego network.

## Results

### Patient Transfers among LTCFs

**Figure 1:**
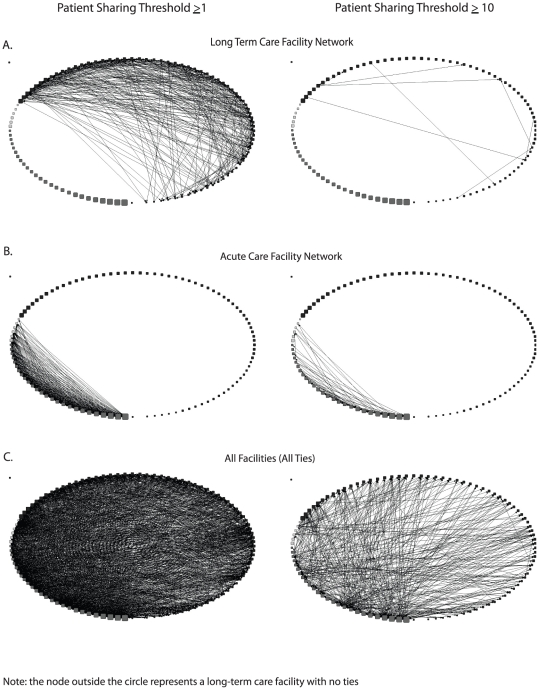
Sociograms of Orange County Healthcare Facility Network at Two Patient Sharing Thresholds. A) Long-term Acute Care Facility (LTCF) Network. B) Acute Care Facility Network. C) All Facilities.

LTCF facility-only network sociograms (Figure 1A) and measures (Table 3) show that a number of LTCFs directly transfer patients to one another. Of all the possible pairs of LTCFs, 8.3% directly transferred at least one patient to each other over the course of the year (network density = 8.3%). Over the course of a year, only six of 72 LTCFs did not send any patients to any other LTCF (out-degree = 0), and only five did not receive any patients directly from other LTCFs (in-degree = 0). Many LTCFs had unequal patient transfer relationships; 29 LTCFs had greater in-degrees than out-degrees, i.e., received patients from a greater number of different LTCFs than the number of LTCFs to which they sent patients. Conversely, 32 had out-degrees greater than in-degrees, i.e., sent patients to greater number of different LTCFs than the number of different LTCFs from which they received patients.

**Table 3:** General Network Measures of Healthcare Facility Network at Patient Transfer Thresholds of ≥1 and ≥10.

Social Network Measure	Long-Term Care Facilities Network		Acute Care Facilities Network		All Facilities	
	≥1	≥10	≥1	≥10	≥1	≥10
**Number of Ties**	426	9	429	63	2,379	536
**Density**	8.3%	0.2%	43.2%	6.4%	22.2%	5.0%
**Reciprocity**	18.7%	0.0%	45.4%	12.5%	41.9%	40.3%
**Network Diameter (Number of Facility Pairs)**	5 (23)	2 (1)	3 (67)	9 (1)	4 (11)	7 (6)
**Facilities with a Geodesic Distance of 1**	10.3%	90.0%	44.6%	16.2%	22.9%	6.6%
**Betweenness** *	44 (0–446)	0 (0–1)	10 (0–75)	225 (0–1,067)	27 (0–881)	38 (0–1,067)
**Out-degree** *	5 (0–21)	0 (0–4)	15 (2–24)	2 (0–7)	17 (0–83)	3 (0–26)
**In-degree** *	5.5 (0–16)	0 (0–2)	13 (0–25)	1 (0–7)	20.5 (0–66)	4 (0–23)

*Median (Range).

As Table 3 demonstrates, a much smaller number of facilities transferred or received at least 10 patients to or from another long-term care facility over the course of a year. Sixty-six LTCFs did not send ≥10 patients to other LTCFs, and 65 LTCFs did not receive ≥10 patients from other LTCFs (data not shown). Only one LTCF both sent and received ≥10 patients in the LTCF network, and the maximum number of patients transferred by an LTCF to another LTCF was 34. The ≥10 patient threshold network included no reciprocated connections, i.e., no facility both transferred and received more than 10 patients over the course of a year. Nine LTCFs had 0 betweenness, and seven had a betweenness >200.

### Patient Transfer Networks Among Acute Care and Long-Term Care Facilities

Figure 1B shows the sociograms and Table 3 shows social network measures for the acute care facilities only network at both patient transfer thresholds. At a threshold of ≥1 patient transfer, all acute care facilities sent patients to at least one other (out-degrees ≥1), and all but one (an LTAC) received patients from at least one other acute care facility. Only one acute care facility was not a necessary intermediary between any pair of acute care facilities (betweenness of 0). Using the threshold of ≥10 patients, ten acute care facilities (six hospitals and four LTACs) did not send patients to at least one other (out-degrees ≥1), and 11 acute care facilities (seven hospitals and four LTACs) did not receive any patients from other acute care facilities. As seen in Figure 1B, three LTACs have no connections at the ≥10 patient threshold. Two of the three share patients across several facilities without a single facility sharing more than 10 patients; the remaining LTAC (children's facility) shares very few patients overall.

Adding LTCFs to the acute care facility network added 1,524 ties between the acute care facilities and LTCFs (≥1 patient shared). Considering all patient transfers in the network (i.e., acute care to acute care, LTCF to LTCF, acute care to LTCF, and LTCF to acute care), there were 2,379 ties. The network was highly heterogeneous at the ≥1 patient transfer threshold (Table 3); it was much more loosely connected at the ≥10 patient transfer threshold. Figure 1C shows sociograms for the LTCF and acute care facility network (i.e., all facilities). As can be seen, LTCFs have several connections and constitute a large portion of patient transfers.

Centrality measures (Table 3) further demonstrated the heterogeneity of the connections. At the ≥1 patient transfer threshold, all but two facilities (both LTCFs) sent patients to other facilities, and only one facility (an LTCF) did not directly receive patients from any other facility. Most facilities had unequal patient transfer relationships; 34 facilities (22 hospitals, six LTACs, and six LTCFs) had out-degrees greater than in-degrees and 65 facilities (two hospitals and 63 LTCFs) had greater in-degrees than out-degrees. Betweenness showed differences in the facilities' involvement in the network. Three LTCFs had 0 betweenness, while seven hospitals and one LTAC had a betweenness >300.

At the ≥10 patient transfer threshold, 16 facilities (2 hospitals, 3 LTACs, 11 LTCFs) had an out-degree of 0, and 11 facilities (2 hospitals, 4 LTACs, 5 LTCFs) did not receive ≥10 patients from any other facility (in-degree = 0). Most facilities had unequal patient transfer relationships. Twenty-nine facilities (21 hospitals, 2 LTACs, and 6 LTCFs) had out-degrees greater than in-degrees and 59 facilities (4 hospitals, 1 LTAC, and 54 LTCFs) had greater in-degrees than out-degrees. Twenty-five facilities (3 hospitals, 4 LTACs, 18 LTCFs) had 0 betweenness, while 10 facilities (9 hospitals, 1 LTCFs) had a betweenness >500.

**Figure 2:**
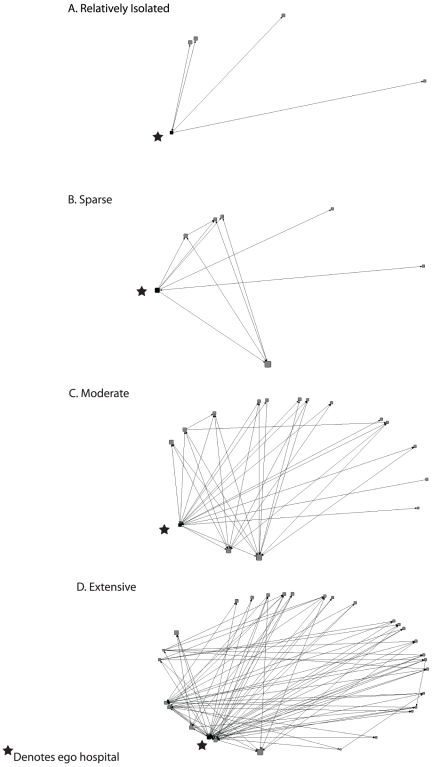
Acute Care Facility Ego Networks at the ≥10 Patient Sharing Threshold. A) Relatively Isolated Facility. B) Sparsely Connected Facility. C) Moderately Connected Facility. D) Extensively Connected Facility.

Table 4 summarizes the ego network measures of the acute care facilities both with and without connections to the LTCFs. As shown, the average size, number of ties, and betweenness of an acute care facility's ego network greatly increases when all ties are considered, and the network density decreases. Table 4 also shows that there was considerable variability in the different acute care facility ego networks considering all ties. The OC healthcare ego networks (N≥10) identified relatively isolated hospitals (Figure 2A), as well as sparsely, moderately, and extensively connected hospitals (Figure 2B, 2C, and 2D, respectively). Four facilities (1 hospital and 3 LTACs) were completely isolated (i.e., had no direct ties to other facilities) in the healthcare facility network (only one more was connected by the addition of LTCFs to the network). The relatively isolated hospital (Figure 2A) was completely isolated from the other acute care facilities but was connected to four LTCFs through direct patient transfers. The sparsely connected hospital (Figure 2B) was connected to one hospital and five LTCFs, which were connected by six ties not involving the ego. The moderately connected hospital (Figure 2C) was connected to 15 facilities with 30 interconnecting ties (not including those to and from the ego hospital). Figure 2D shows an extensively connected hospital, which had 80 ties not including those to and from the ego hospital and included 28 facilities.

**Table 4:** Ego Network Measures for Acute Care Facilities at the Patient Transfer Threshold of ≥10.

Ego Network Measure	Acute Care Facilities		All Facilities*	
	Mean (SD)	Median (Range)	Mean (SD)	Median (Range)
**Size**	3.5 (2.7)	3.5 (0–8)	13.4 (9.1)	14.5 (0–29)
**Ties**	2.25 (2.6)	1 (0–9)	30.4 (30.5)	23.5 (0–122)
**Density**	16.07% (20.8)	12.5% (0–100)	12.8% (9.8)	11.9% (0–40)
**Betweenness**	3.29 (4.5)	0.5 (0–15.8)	103.4 (129.9)	58.1(0–428.4)

Note: SD is standard deviation.

*Change in acute care facility ego network measures when LTFCs are added to the network.

### LTCFs Connecting Acute Care Facilities

LTCFs connected many acute care facilities with each other that otherwise were not connected. Comparing the various sociograms of Figure 1 shows that direct patient transfers between hospitals comprises only a fraction of the inter-facility patient transfers; the ratio of acute care facility network ties to ties to or from LTCFs was 28.1% and 13.6% at thresholds of ≥1 and ≥10 patients shared, respectively.

At the ≥10 patient transfer threshold, 604 of the directional (i.e., non-symmetric) acute care facility pairs were not associated by patient transfers in the acute care facility network (i.e., there were 604 instances where a hospital either did not send or receive at least 10 patients to or from another hospital). Adding LTCFs to the network resulted in the formation of 289 associations, decreasing the number of unassociated acute care facility pairs to 315.

### Correlation Between Patient Transfers and Geographic Distance

Geodesic distance (i.e., the shortest number of inter-facility ties that connect one facility to another) correlated somewhat with the geographic distance between the facilities, although it was well below 100%. The correlation coefficient was 0.24 (≥1 patient threshold), suggesting that closeness in facility geographic distance does not explain all of the patient sharing relationships and that many distant facilities share patients.

## Discussion

Understanding the network of patient transfers may be important for multiple reasons. Patients can carry the ramifications of previous healthcare facility stays. For example, previous stays can determine a patient's course of treatment [7], [8] (e.g., what medications and procedures the patient has or continues to receive), habits [7], [8] (e.g., what smoking-cessation or other behavioral modification programs the patient may have undergone), expectations and information [7], [8] (e.g., what education and counseling the patient has received), disease evolution [7] (e.g., how aggressively blood pressure is controlled and monitored), and infectious disease carriage status [9], [10] (e.g., what is the patient's risk for methicillin-resistant *Staphylococcus aureus*). This may be especially true for LTCFs, which host patients for longer time periods and therefore may have a greater impact on patients for certain things (e.g., patient habits or infectious pathogen colonization status).

In this way and others, LTCFs could be important yet under-recognized members of the acute care facility network. When hospitals institute policies and programs, how often do they consider the LTCFs to which they are connected and the other facilities to which these are connected? For example, does a hospital's infection control program account for the infection control programs of all connected LTCFs? Does a hospital's formulary consider the formularies of all other LTCFs? Does a hospital coordinate its behavioral change programs (e.g., smoking cessation or dietary counseling) with those of connected facilities? While studies have looked at coordinating between facility transfers (including improved communication of advanced directives and medication reconciliation) for individual patients [11], [12], [13], [14], large scale coordination among multiple facilities does not always occur [12], [13], [15], [16]. In fact, as our study demonstrates, acute care facilities may be unknowingly connected to each other through long-term care intermediaries. The inter-connectedness among a community's healthcare facilities has wide-ranging implications for patient safety, health policy, and law.

The substantial number of transfers occurring between acute and long-term care facilities and between LTCFs are likely the result of different phenomena [12]. Patients may be changing insurance policies which affect which facilities they may utilize. A patient's changing health status (e.g., disease exacerbations or improvements and new diseases) may necessitate his or her movement to a facility with the personnel, orientation, service, or size to handle a new type of care [17]. Periodic treatment may also require occasional transfers. Patients and their families may prefer another facility.

Certain LTCFs may be particularly interconnected with acute care facilities and could serve as key targets for interventions. For example, it may be especially important to ensure that an extensively connected LTCF has effective infection control, disease management, patient education, and treatment policies. With limited resources, public health officials and other policy makers may want to focus resources and efforts first on a few highly interconnected facilities to enact change among all LTCFs.

### Limitations

Our study has several limitations. Although most patients receiving healthcare in OC stay within the county to receive care, some do cross county lines. For LTCFs, 83.4% of transfers between LTCFs and 95.8% of transfers to acute care facilities were within the county; for OC acute care facilities, 94.8% of transfers to LTCFs and 87% of acute care transfers were to facilities within the county. Lastly, while a large diversity of hospital types and sizes were represented, the generalizability of our findings to other counties remains unclear.

### Conclusions

Our study had several findings: (1) many LTCFs directly transfer patients to each other; (2) LTCFs connect many acute care facilities with each other that are not otherwise directly connected to each other; (3) these connections can occur over many miles. These findings suggest that acute care facilities should account for connections with and among LTCFs. Understanding the acute and long-term care social network can help hospital administrators, public health officials, and other key decision makers plan and implement interventions.
